# All-SAM interfacial architecture for perovskite solar cells without charge transport materials

**DOI:** 10.1039/d5sc06906h

**Published:** 2026-02-24

**Authors:** Zhanhao Hu, Nao Saito, Masashi Ikegami, Naoyuki Shibayama, Tsutomu Miyasaka

**Affiliations:** a Graduate School of Engineering, Toin University of Yokohama Yokohama Kanagawa Prefecture 225-8503 Japan miyasaka@toin.ac.jp shibayama@toin.ac.jp

## Abstract

State-of-the-art perovskite solar cells (PSCs) employ a multilayer device structure, incorporating a combination of charge transport layers and interfacial modifications to achieve efficient charge extraction. However, simplifying the device structure is highly desirable for cost-effective mass production. One approach is to integrate multiple functions into a single monolayer, replacing the multilayer structure. To explore this concept, we propose a device architecture where a combination of p-type and n-type self-assembled monolayers (SAMs) is employed to construct the hole-extraction and electron-extraction interfaces in a PSC without charge transport layers. The resulting device successfully establishes charge selectivity, achieving a substantial photovoltaic output and promising stability. This fully SAM-based interfacial architecture presents a promising strategy for making efficient solar cells with a minimum demand of materials and processes. The proposed device architecture can be applied to other types of thin-film devices and introduces a new approach of designing interfaces at the molecular level.

## Abstract 2

State-of-the-art perovskite solar cells employ complex multilayer interfaces composed of hole and electron transport layers and additional interlayers. Here, we replace these multilayer interfaces with a single self-assembled monolayer (SAM).

## Graphical abstract

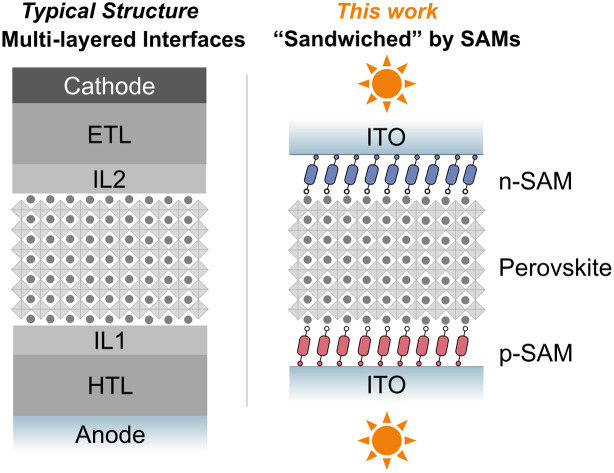

## Introduction

In thin-film electronic devices, heterojunction interfaces where charge carriers encounter energetic and electronic discontinuities are often sites of significant physical processes, both desirable and undesirable. Designing an interface with optimal efficiency requires careful consideration of multiple factors. In a perovskite solar cell (PSC), an ideal interface should maximize the extraction efficiency of photogenerated carriers while minimizing energy losses.^1–3^ To achieve this, the interface must possess high charge mobilities, minimal trap states and appropriate energy levels. However, meeting all of these diverse requirements poses substantial challenges. As a result, complex interfacial structures comprising multiple layers, each tailored to fulfill one or a set of the requirements, are generally adopted in state-of-the-art PSCs. A typical device may incorporate one or more hole transport layers, one or more electron transport layers, and additional passivation treatments at the heterojunction interfaces.^4^

**Fig. 1:**
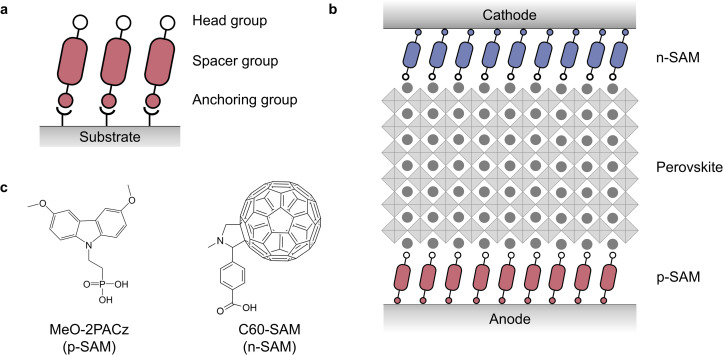
(a) The components of a SAM molecule and its self-assembly on the substrate. (b) The charge-transport-layer free device architecture incorporating SAMs at the interfaces. (c) The molecular structures of the p-SAM (MeO-2PACz) and n-SAM (C_60_-SAM) used in this experiment.

However, the multilayer device structure and its complex deposition processes can increase costs, lower yields in large-scale production and reduce the recyclability of the solar cells.^5–7^ Therefore, minimizing the number of interfacial layers and treatments is always desirable. One approach to simplify the interfaces is to reproduce functions of the multilayer interfaces with a single molecule. The molecule is composed of multiple segments, each of which fulfills one or a set of the requirements to form a desirable interface. Such an approach is well-established in molecular electronics where molecules with precisely engineered multifunctionalities are designed to replicate the functions of bulk materials in a conventional device.^8^ One class of these molecules is those that readily form an ordered self-assembled monolayer (SAM) through bonding with the adjacent layers.^9–11^ A typical SAM molecule consists of three components including an anchoring group, a spacer group and a head group (Fig. 1a). These components can be tailored to control properties such as charge transport, energy level alignment, interfacial passivation and surface energy, offering a rich versatility and providing a template to design interfaces from bottom-up.^9,12^ For their applications in PSCs, Snaith and co-workers reported using SAMs to modify the electron transport layer of TiO_2_ in 2014.^13^ In 2019, the Albrecht group developed carbazole-based SAMs to replace the hole transport layer of poly[bis(4-phenyl)(2,4,6-trimethylphenyl)amine].^14^ Driven by the development of a wide variety of SAMs,^12–15^ both the power conversion efficiency (PCE) and device stability have been significantly improved, with efficiencies beyond 27% reported in recent studies.^15^ Nonetheless, due to challenges such as dissolution of the perovskite layer during solution-based deposition of SAMs on top, state-of-the-art PSCs still rely on charge transport layers either on both sides of the electrodes or on one side.^13–21^ Commonly used charge transport materials such as TiO_2_ and NiO_*x*_ require high-temperature annealing which is incompatible with flexible substrates.^22^ The organic hole transport layers such as 2,2′,7,7′-tetrakis-(*N*,*N*-di-4-methoxyphenylamino)-9,9′-spirobifluorene (Spiro-OMeTAD) are sensitive to moisture and high temperature, leading to accelerated device degradation.^23^ Therefore, eliminating charge transport layers without compromising device efficiency is highly desirable.

In this study, we fully replace charge transport layers at both the anode and cathode with a single layer of a p-type SAM (p-SAM) and an n-type SAM (n-SAM) respectively (Fig. 1b). Indium tin oxide (ITO) is adopted for both electrodes to ensure any device performance can be attributed to the sole contribution from the SAMs. The resultant device exhibits a successful establishment of an asymmetric charge injection. More promisingly, a photovoltaic output with an open-circuit voltage (*V*_OC_) and short-circuit current density (*J*_SC_) close to those of state-of-the-art PSCs is achieved.

## Results and discussion

### Device structure and fabrication

Our device has a simple structure of anode/p-SAM/perovskite/n-SAM/cathode (Fig. 1b). Specifically, ITO is used for both electrodes to ensure that establishment of charge selectivity can be attributed to the SAMs exclusively. Furthermore, a hydroxylated surface can be easily formed by treating ITO with UV-Ozone which provides covalent bonding sites to SAM molecules that include phosphonic acids (–PO(OH)_2_) or carboxylic acids (–COOH) as the anchoring groups. [2-(3,6-Dimethoxy-9*H*-carbazol-9-yl)ethyl]phosphonic acid (MeO-2PACz) is employed for the p-SAM, and 4-(1′,5′-dihydro-1′-methyl-2′*H*-[5,6]fullereno-C_60_-Ih-[1,9-*c*]pyrrol-2′-yl)benzoic acid (C_60_-SAM) is employed for the n-SAM (Fig. 1c). Both molecules were well-studied and commercially available.^13,14,24^

**Fig. 2:**
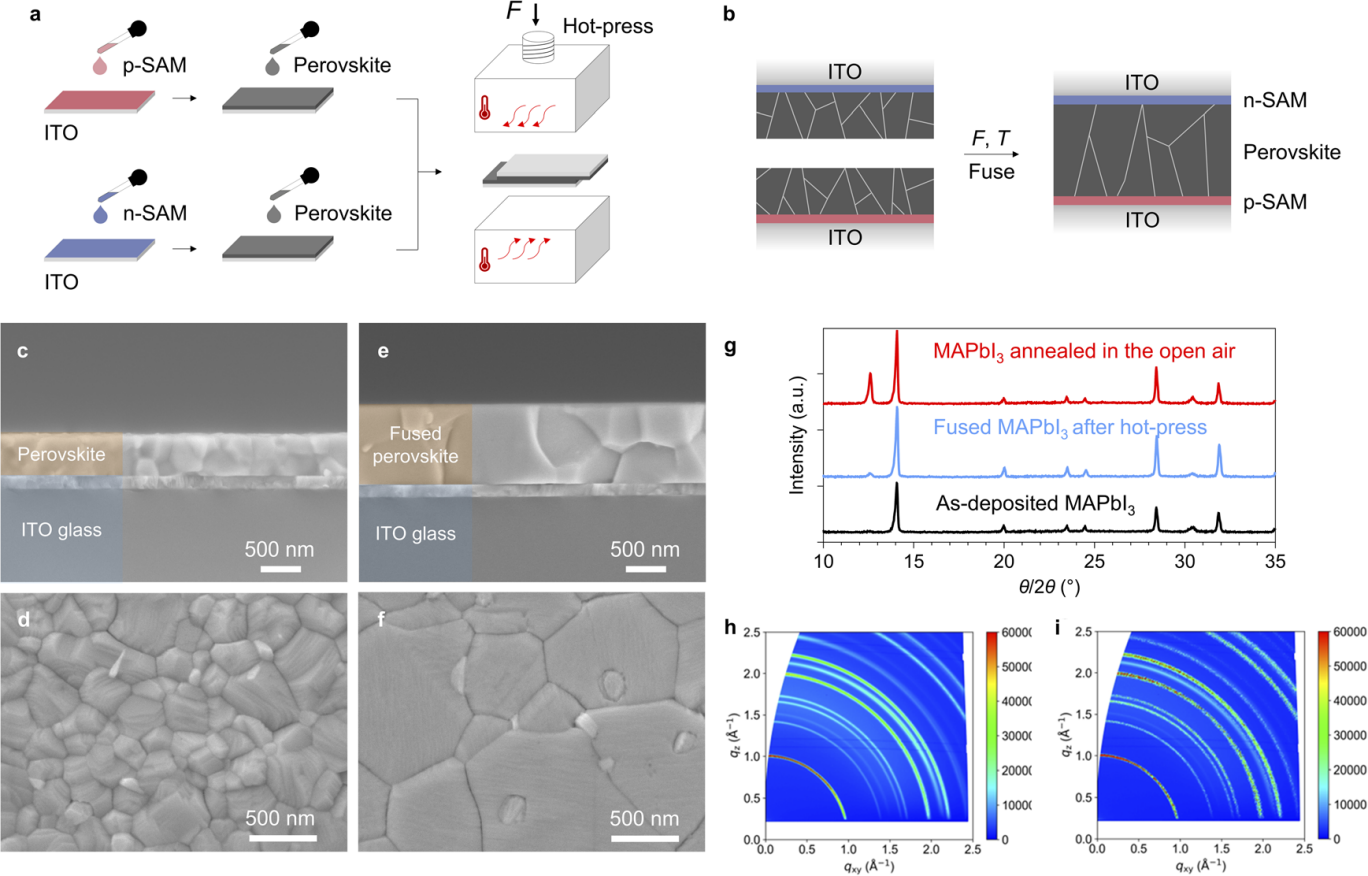
(a) The device is fabricated by depositing the perovskite on p-SAM-modified ITO and n-SAM-modified ITO separately, followed by thermal annealing under pressure in a hot-press. (b) The two perovskite films fuse into one single uniform layer after the hot-press process. (c) The cross-section of the perovskite before hot-press. (d) The surface of the perovskite before hot-press. (e) The cross-section of the perovskite after hot-press (the upper ITO substrate is removed). (f) The surface of the perovskite after hot-press (the upper ITO substrate is removed). (g) XRD patterns of the as-deposited perovskite, the fused perovskite after the hot-press process and the perovskite thermally annealed in the open air. (h) GIWAXS 2D patterns of MAPbI_3_ before hot-press. (i) GIWAXS 2D patterns of MAPbI_3_ after the hot-press process.

To fabricate the device, p-SAM and n-SAM are separately spin-coated onto two pieces of ITO substrates (Fig. 2a). The deposited SAMs are thermally annealed and washed with their respective solvents to remove excess unbound molecules. Subsequently, the perovskite layer of methylammonium lead iodide (MAPbI_3_) is spin-coated onto the p-SAM and n-SAM, respectively. MAPbI_3_ is selected due to its well-studied properties and widespread use as a benchmark perovskite composition. The two samples are then stacked with the perovskite layers in contact and annealed at 150 °C under a pressure of about 1.2 × 10^7^ Pa in a hot-press for 20 min (Fig. S1). After this process, the two perovskite layers fuse to form one single layer with high uniformity (Fig. 2b). To examine the perovskite layer before and after fusion by scanning electron microscopy (SEM), perovskite layers were prepared on a bare ITO and an ITO/p-SAM substrate respectively. Fig. 2c and d show the cross-section and surface of the perovskite layer (approximately 500 nm thick) on ITO before hot-press. The two stacks of ITO/MAPbI_3_ and ITO/p-SAM/MAPbI_3_ were then hot-pressed to form ITO/MAPbI_3_/p-SAM/ITO. The upper ITO substrate was subsequently removed to enable SEM imaging. Owing to the weaker adhesion of MAPbI_3_ on p-SAM compared to its adhesion on bare ITO, the perovskite film delaminates from the ITO/p-SAM interface.^25^ The surface and cross-section of the revealed perovskite (approximately 1000 nm thick) are shown in Fig. 2e and f. The perovskite layer is seamlessly fused in the vertical direction with no observable gaps at the laminated interface (a cross-section image at lower magnification is provided in Fig. S2). Notably, many perovskite grains extend across the full thickness of the layer.

The fusion of two perovskites under heat and pressure can be explained by Ostwald ripening of ionic crystals.^26–28^ Mass transport between grains merges the two unconnected layers, resulting in grain growth after hot-press (Fig. 2c–f). In the meantime, the sequential process of thermally induced perovskite decomposition and its reversible formation in the closed system could also have played a role.^29,30^ For MAPbI_3_ annealed in the open air at 150 °C for 20 min, the X-ray diffraction peak of the decomposed species of PbI_2_ (12.6°) increased rapidly indicating accelerated degradation of MAPbI_3_ at this temperature (Fig. 1g). However, for the hot-pressed perovskite annealed at the same temperature, the PbI_2_ peak is largely suppressed and the crystal structure of MAPbI_3_ is maintained. Grazing-incidence wide-angle X-ray scattering (GIWAXS) shows consistent results, indicating that both the crystal quality and crystallite size remain intact after the hot-press process (Fig. 2h and i). The 1D integrated profiles are provided in Fig. S3, and the calculated crystallite sizes are summarized in Table S1. Furthermore, UV-vis absorption spectra of the MAPbI_3_ before and after hot-press exhibit identical bandgaps of approximately 1.60 eV (Fig. S4). We therefore supposed that the closed system created by the intimate contact between the two pieces of ITO substrates prevents the decomposed gaseous species from escaping far from the film and thereby enables the back reaction route to reform the perovskite. Fusion of two perovskites and the preservation of their crystalline quality through the hot-press process have also been confirmed by several other groups.^31–34^

It is worth noting that the ultra-simple device architecture proposed here has the potential to reduce manufacturing costs compared with conventional multilayer devices. Although the exact cost depends on factors such as material prices, deposition processes, device yield, and other variables, a comparison limited to the interfacial materials indicates that the material cost of the all-SAM interface architecture can be only a fraction of that of conventional charge-transport materials (cost analysis is provided in Tables S2 and S3 in the SI). Furthermore, by eliminating the need to selectively dissolve and recycle multiple transport layers, the all-SAM interface can facilitate easier device recycling and is therefore compatible with a greener, circular economy.^6,7^

### Electrical and photovoltaic characteristics

**Fig. 3:**
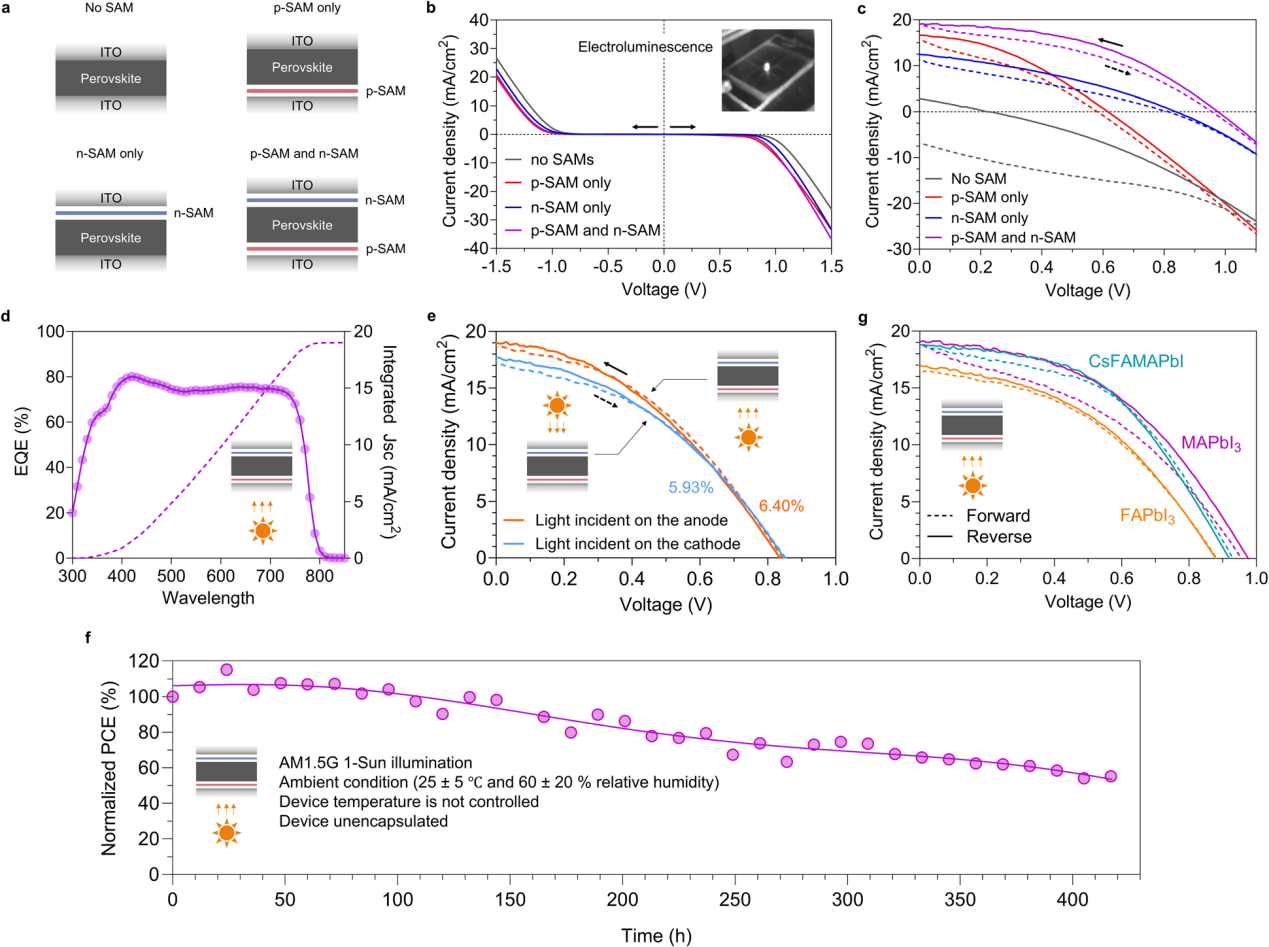
(a) The four device structures under investigation. (b) Dark *J*–*V* of the four types of devices with MAPbI_3_ as the perovskite layer. The picture shows the electroluminescence of the device with both the p-SAM and n-SAM operated at about 15 V. (c) Photovoltaic characteristics of the four types of devices with MAPbI_3_ as the perovskite layer. (d) The EQE spectrum (left *y*-axis) of the device ITO/p-SAM/MAPbI_3_/n-SAM/ITO. The integrated current density is plotted to the right *y*-axis. (e) Photovoltaic characteristics of the device ITO/p-SAM/MAPbI_3_/n-SAM/ITO with light incident on the anode and cathode respectively. (f) Device (unencapsulated) stability of ITO/p-SAM/MAPbI_3_/n-SAM/ITO under maximum power point tracking. (g) Photovoltaic characteristics of the devices (ITO/p-SAM/perovskite/n-SAM/ITO) with MAPbI_3_, FAPbI_3_ and CsFAMAPbI as the perovskite layers.

We fabricated four types of devices to investigate their electrical and photovoltaic characteristics (Fig. 3a): (1) the device with no SAMs and thereby has a symmetric structure of ITO/perovskite/ITO; (2) the device with the p-SAM, *i.e.*, ITO/p-SAM/perovskite/ITO; (3) the device with the n-SAM, *i.e.*, ITO/perovskite/n-SAM/ITO; and (4) the complete device with both the p-SAM and n-SAM, *i.e.*, ITO/p-SAM/perovskite/n-SAM/ITO. Dark current density–voltage (*J*–*V*) characteristics from 0 V to 1.50 V, and subsequently from 0 V to −1.50 V are given in Fig. 3b. Unsurprisingly, the symmetric device with no SAMs exhibits symmetric current injection characteristics under both positive and negative bias. The turn-on voltage is observed at 1.02 V and −1.02 V, with the current density reaching approximately 26 mA cm^−2^ at both 1.50 V and −1.50 V. By adding the p-SAM on one side of the device, charge injection under positive bias is slightly enhanced, reducing the turn-on voltage to about 0.88 V. Conversely, charge injection at negative bias is suppressed, increasing the turn-on voltage to −1.13 V. Similarly, the device with only the n-SAM shows an enhancement in charge injection under positive bias yielding a turn-on voltage of 0.96 V. Injection under negative bias is suppressed with a turn-on voltage of −1.08 V. For the complete device incorporating both the p-SAM and n-SAM, the turn-on voltage is 0.92 V under positive bias and −1.13 V under negative bias. The device achieves a highest current density of 36.8 mA cm^−2^ at 1.50 V, while exhibiting a relatively suppressed current density of 20.9 mA cm^−2^ at −1.50 V. Dark *J*–*V* curves obtained by a continuous forward and reverse scan are provided in Fig. S5, where varying hysteresis is observed indicating different charge injection barriers among the four types of devices. As a result, despite that the rectification ratio is modest, an asymmetric charge injection is successfully established with the insertion of SAM at the interfaces, and the best performance is obtained in the device incorporating both the p-SAM and n-SAM. Notably, clear electroluminescence is observed at positive bias (Fig. 3b), indicating that our device architecture is also applicable to construct light-emitting diodes.

It is then interesting to investigate whether such a simple device structure can produce notable asymmetric charge extraction under light illumination, *i.e.*, a photovoltaic output. Since ITO glass is used for both electrodes, the device can be illuminated from either side. For consistent comparison, AM1.5G 1-Sun light illumination (100 mW cm^−2^) was directed onto the anode side, while a black sheet was placed on the cathode side to prevent reflected light from re-entering the device. The photocurrent is shown in Fig. 3c. As expected, the symmetric device without SAMs exhibits symmetric *J*–*V* characteristics with no photovoltaic output (a wider scan from −1.2 V to 1.2 V is given in Fig. S6). The mismatch of the forward and reverse scans can be attributed to the hysteresis.^35,36^ Upon adding the p-SAM on one side of the device, a substantial photovoltaic output arises yielding a *V*_OC_ of 0.62 V, a *J*_SC_ of 16.6 mA cm^−2^ and a fill-factor (FF) of 37.6% (the external quantum efficiency (EQE) spectrum is provided in Fig. S7). This corresponds to a PCE of 3.85%. Similarly, adding the n-SAM on one side of the device achieves a PCE of 3.56% with a *J*_SC_ of 12.5 mA cm^−2^, a *V*_OC_ of 0.84 V and a FF of 34.1% (the EQE spectrum is provided in Fig. S7). When both the p-SAM and n-SAM are incorporated into the device, a promising PCE of 8.40% is achieved which is higher than those of both of the devices incorporating only the p-SAM and only the n-SAM (statistical performance data are provided in Fig. S8). All key parameters show improvement. Notably, *J*_SC_ reaches 19.1 mA cm^−2^ which is approximately 81% of the value previously reported for the state-of-the-art PSCs based on MAPbI_3_.^37^ The integrated current density (19.0 mA cm^−2^) from the EQE spectrum matches the *J*_SC_ as shown in Fig. 3d. Meanwhile, *V*_OC_ reaches 0.98 V which is approximately 88% of the value reported for the state-of-the-art MAPbI_3_ device.^37^ However, FF remains relatively low at 45.2% which will be discussed in the next section.

Our results demonstrate that the device architecture with SAMs alone at the interfaces is sufficient to achieve a substantial photovoltaic output. To the best of our knowledge, charge-transport-layer-free perovskite solar cells with SAMs were previously reported by Udo Bach *et al.*, who deposited two different types of SAMs onto horizontally placed gold electrodes in a back-contact device structure.^38^ MAPbI_3_ was directly deposited onto the SAM-modified electrodes, yielding a *V*_OC_ of 0.56 V, *J*_SC_ of 11.4 mA cm^−2^, FF of 40.5% and PCE of 2.59%. In comparison, our results using a sandwiched device structure show improvements across all key parameters, suggesting more efficient charge extraction in our architecture.

It is worth noting that the use of highly transparent ITO glass as both the electrodes, combined with a fully charge-transport-layer-free structure that minimizes parasitic absorption, enables our device to function as an ideal bifacial solar cell. A very small performance difference is observed when light is incident on the anode compared to the cathode (Fig. 3e). A bifaciality factor of 93% is obtained which is on par with the best bifacial silicon solar cells, and is among the highest achieved in PSCs.^39,40^

Furthermore, the device exhibited a promising stability without additional encapsulation. 80% of its initial PCE is maintained after about 250 hours under continuous AM1.5G light illumination in ambient air (25 ± 5 °C and 60 ± 20% relative humidity) in accordance with the ISOS-L-1 protocol^41^ (Fig. 3f). The two ITO glass electrodes act as a form of self-encapsulation which significantly retards perovskite degradation. Decomposed PbI_2_ was observed to proceed from the device edges indicating oxygen and water infiltration primarily through the gap between the two ITO electrodes (Fig. S9). This inherent stability could eliminate the need for high quality encapsulation, thereby reducing the associated costs.

The ultra-simple device structure can be extended to other perovskite formulations such as formamidinium (FA)-based perovskites, which have higher thermal stability compared to MAPbI_3_. Devices incorporating FAPbI_3_ and Cs_0.05_FA_0.85_MA_0.1_PbI_3_ (CsFAMAPbI) were fabricated where the perovskites were prepared through a modified recipe (provided in SI) from previous reports.^42,43^ The crystal quality after hot-press remains intact as confirmed by the GIWAXS 2D patterns (Fig. S10), 1D integrated profiles (Fig. S11) and crystallite calculation (Table S1). The photovoltaic performance is compared in Fig. 3g and Table S4. Both FAPbI_3_ and CsFAMAPbI exhibit promising photovoltaic outputs, reaching a maximum PCE of 7.34% and 8.22% respectively. Notably, *V*_OC_ as large as 1.01 V is observed with FAPbI_3_, and a negligible hysteresis (forward-to-reverse PCE ratio of 98.8%) is achieved with CsFAMAPbI.

### Energy level alignment

**Fig. 4:**
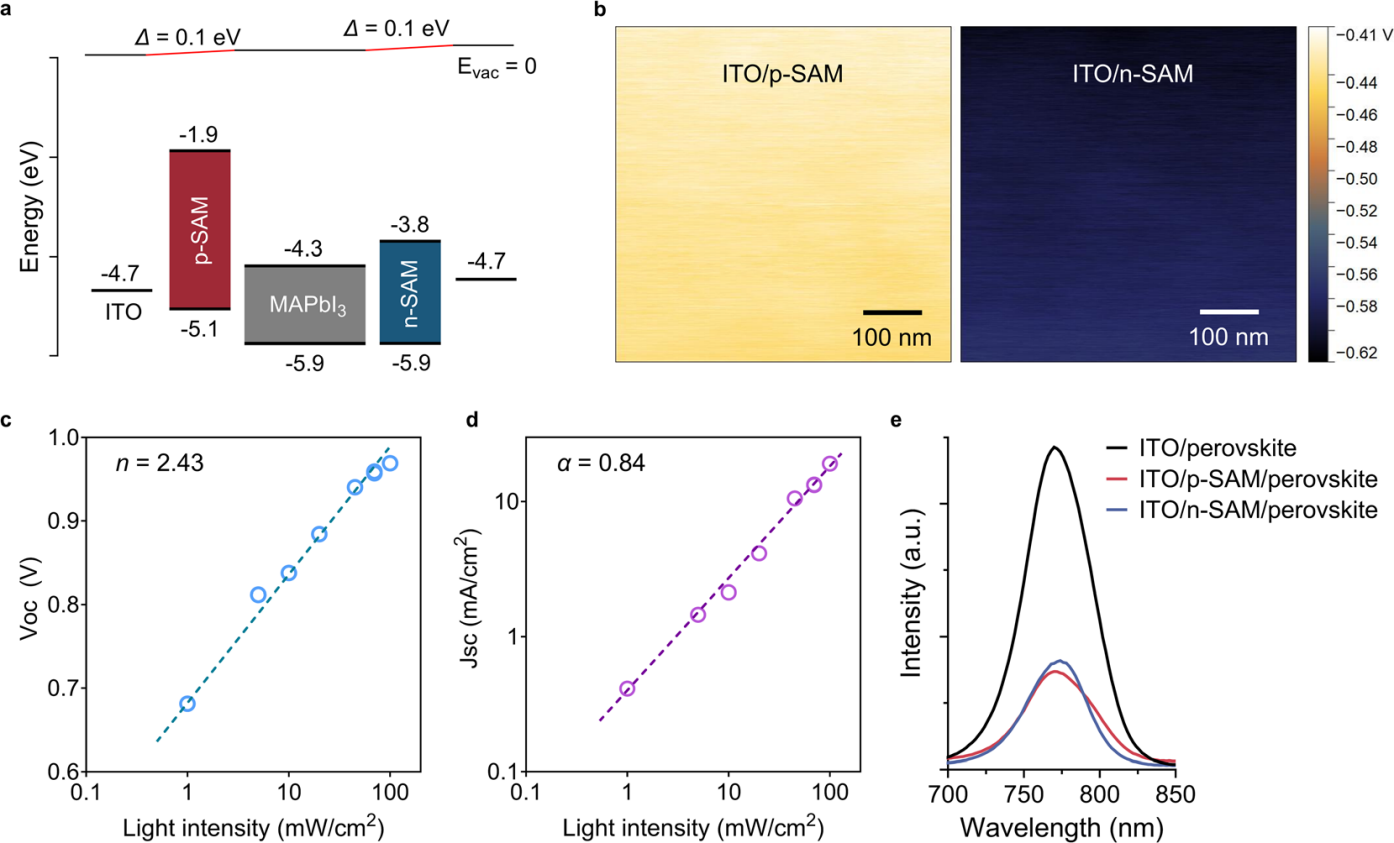
(a) The energy diagram of the complete device. (b) Surface potential of the p-SAM-coated ITO and the n-SAM-coated ITO. (c) Light-intensity dependent *V*_OC_, (d) light-intensity dependent *J*_SC_, and (e) photoluminescence of MAPbI_3_ deposited on the bare ITO, p-SAM-coated ITO and n-SAM-coated ITO.

To better understand the establishment of electrical polarity from SAMs, the energy level alignment was investigated by ultraviolet photoelectron spectroscopy (Fig. S12). The bandgaps were calculated from UV-vis spectroscopy (Fig. S13). The valence band maximum (VBM) and conduction band minimum (CBM) of MAPbI_3_ are from previous reports.^44,45^ The resulting energy diagram is presented in Fig. 4a. The energy levels are consistent with the literature.^14,44,46^ The ITO substrate exhibits a work-function of 4.7 eV. Adding the p-SAM and n-SAM slightly modifies the surface work-function to 4.8 eV and 4.6 eV respectively. The modest difference of 0.2 eV indicates a limited built-in electric field within the perovskite layer in contrast to state-of-the-art PSCs that employ electrodes with significantly larger work-function disparity.^47–49^ The areal surface potentials of p-SAM- and n-SAM-modified ITO, measured by Kelvin probe force microscopy, are shown in Fig. 4b, revealing uniform SAM coverage. Regarding the energy barriers, at the anode side, the highest occupied molecular orbital (HOMO) of the p-SAM is located at −5.1 eV forming no barrier to hole extraction from the VBM of MAPbI_3_ (−5.9 eV). However, the hole injection barrier of approximately 0.8 eV remains relatively large which could have induced energy loss and thereby a lower *V*_OC_ in the p-SAM-only device compared to the n-SAM-only device (Fig. 3c). The lowest unoccupied molecular orbital (LUMO) of the p-SAM is located at −1.9 eV, which can effectively block electron leakage from the CBM of MAPbI_3_ (−4.3 eV). At the cathode side, the HOMO of the n-SAM is positioned at −5.9 eV, which may lead to insufficient hole blocking. Additionally, the LUMO of the n-SAM is located at −3.8 eV, forming an energy barrier of approximately 0.5 eV from the CBM of MAPbI_3_. This barrier is not insurmountable, but it could impede efficient electron extraction leading to a smaller *J*_SC_ in the n-SAM-only device compared to the p-SAM-only device (Fig. 3c). As a result, the energy level alignment of the device is far from ideal, which may contribute to retarded charge extraction under positive bias, leading to a relatively small FF (Fig. 3c, and Table S4).^50^ In addition, as reported in previous studies, the coverage of SAMs on the substrates is incomplete, resulting in insufficient passivation and direct contact between the perovskite and the electrodes, where charge recombination occurs.^51–56^ Moreover, the insufficient work-function difference between the electrodes (ITO is used for both the anode and cathode herein) may have hindered photocarrier extraction under forward bias, thereby increasing charge recombination and resulting in a reduced FF.^49,57^ Consistent with the interpretation, a relatively large ideality factor (*n*) of 2.43 is extracted from the light-intensity dependent *V*_OC_ (Fig. 4c), and a slope (*α*) of 0.84 is obtained from the light-intensity dependent *J*_SC_ (Fig. 4d), both indicating substantial non-radiative recombination losses.^58,59^ Nonetheless, the energy diagram still provides an asymmetric energy level alignment to enable favorable extraction of holes to the anode and electrons to the cathode. The selective extraction of holes and electrons is further facilitated by the hole-transporting carbazole moieties in the p-SAM and the electron-transporting fullerene moieties in the n-SAM respectively in the complete device.^9,24,60,61^ Photoluminescence (PL) measurements show a consistent result revealing enhanced charge extraction when the SAM is used at the interface. A significant PL quench is observed for MAPbI_3_ deposited on the p-SAM and n-SAM, compared to MAPbI_3_ coated on the bare ITO substrate (Fig. 4e). It is therefore reasonable to expect that device performance can be further improved through strategies such as optimizing the energy-level alignment between the SAMs and the perovskite, increasing SAM coverage, enhancing the interfacial passivation and increasing the built-in electric field.

## Conclusions

In this study, we replaced the complex, multilayered heterojunction interfaces commonly used in state-of-the-art PSCs with a single layer of p-SAM and n-SAM at the anode and cathode respectively. This ultra-simple device structure free of charge transport layers achieves a *J*_SC_ and *V*_OC_ close to those of state-of-the-art PSCs with a promising stability. More importantly, by employing symmetric transparent ITO electrodes, our results demonstrate that SAMs alone can generate sufficient charge selectivity to produce a substantial photovoltaic output. Our findings introduce a fundamentally new PSC architecture to eliminate the need for bulk charge transport materials in heterojunction devices. This architecture has the potential to circumvent the limitations associated with bulk transport materials, reduce manufacturing costs, and enable easier device recycling. We believe that the device efficiency achieved here can be further improved by developing SAMs that form better energy level alignment with the perovskite. Furthermore, the concept of using molecular monolayers to replace multilayer interfaces could be extended to other devices such as light-emitting diodes and memristors, opening up a new pathway to cost-effective electronics.

## Author contributions

Z. H. conceived and designed the experiments; Z. H. fabricated the samples; Z. H., N. S. and NY. S. performed the characterization with support from M. I.; Z. H. wrote the manuscript; and NY. S. and T. M. supervised the research program. The manuscript was written through the contributions of all authors. All authors have given approval to the final version of the manuscript.

## Conflicts of interest

The authors declare no conflict of interest.

## Supplementary Material

SC-017-D5SC06906H-s001

## Data Availability

The data that support the findings of this study are available in the article and the supplementary information (SI), as well as from the corresponding authors upon reasonable request. Supplementary information is available. See DOI: https://doi.org/10.1039/d5sc06906h.
