# Supplementary material for: All-SAM interfacial architecture for perovskite solar cells without charge transport materials
Source: Chem Sci. 2026 Feb 24;17(12):5869–76. doi: 10.1039/d5sc06906h (PMC12955810; doi:10.1039/d5sc06906h)
Supplement: SC-017-D5SC06906H-s001 [file SC-017-D5SC06906H-s001.pdf]

Supplementary Information (SI) for Chemical Science.
This journal is © The Royal Society of Chemistry 2026

Supplementary Information for

All-SAM Interfacial Architecture for Perovskite Solar Cells without Charge Transport Materials

Zhanhao Hu, Nao Saito, Masashi Ikegami, Naoyuki Shibayama* and Tsutomu Miyasaka*

Graduate School of Engineering, Toin University of Yokohama, Yokohama, Kanagawa Prefecture 225-8503, Japan.
E-mail: miyasaka@toin.ac.jp; shibayama@toin.ac.jp

This PDF file includes:

Materials and Methods
Cost analysis
Figures S1 to S13
Tables S1 to S4

Materials and Methods

Materials

Reagents used in this study include methylammonium iodide (MAI, TCI), methylammonium bromide (MABr, TCI), formamidinium iodide (FAI, Sigma-Aldrich), caesium iodide (CsI, TCI), methylammonium chloride (MACl, Luminescence Technology Corp.), lead iodide (PbI_2, 99.99%, TCI), rubidium chloride (RbCl), [2-(3,6-Dimethoxy-9H-carbazol-9-yl)ethyl]phosphonic acid (MeO-2PACz, TCI), 4-(1',5'-Dihydro-1'-methyl-2'H-[5,6]fullereno-C_{60}-Ih-[1,9-c]pyrrol-2'-yl)benzoic acid (C_{60}-SAM, Sigma-Aldrich), ethanol (Super dehydrated, Wako), isopropanol (IPA, Super dehydrated, Wako), toluene (Super Dehydrated, Wako), *N,N*-dimethylformamide (DMF, Super dehydrated, Wako), dimethyl sulfoxide (DMSO, Super dehydrated, Wako), chlorobenzene (CB, Sigma-Aldrich), tetrahydrofuran (THF, Super dehydrated, Wako), acetone (Wako), octane-1,8-diamine dihydroiodide (ODADI, TCI) and ethyl acetate (Super dehydrated, Wako). All reagents were used as received without further purification. ITO glass substrates (12 Ω/sq, ITO is patterned) were purchased from Yingkou OPV Tech New Energy Co., Ltd.

Device fabrication

ITO glass substrates were cleaned sequentially with detergent, deionized water, acetone and isopropanol by ultrasonic bath. Subsequently, ITO substrates were treated with UV-ozone (ASM401oz, ASUMI GIKEN) for 15 min. To deposit the p-SAM, MeO-2PACz (0.1 mM in ethanol) was spin-coated (Opticoat MS-A100, MIKASA) on the ITO substrate followed by thermal annealing at 100 °C for 10 min. To remove the unbound molecules, ethanol was spin-coated on the deposited p-SAM. Similarly, to deposit the n-SAM, C_{60}-SAM (1 mM in the mixed solvents of CB and THF (1:1 v/v)) was spin-coated on the ITO substrate followed by thermal annealing at 100 °C for 10 min. The film was subsequently washed by its solvent to remove unbound molecules.

To deposit $MAPbI_3$, the precursor was prepared by mixing 465.6 mg PbI_2 and 159 mg MAI in 530 uL DMF and 73 uL DMSO. The precursor was spin-coated on the substrate at 3800 rpm for 40 s. At 10 s from the start of spin-coating, toluene as the anti-solvent was dropped onto the film. The resultant sample was pre-annealed at 60 °C for 5 min and subsequently annealed at 100 °C for 15 min.

For the deposition of $FAPbI_3$, 1.5 M of PbI_2 mixed with 5 molar% RbCl was dissolved in the mixed solvents of DMF and DMSO (9:1 v/v) and spin-coated on the substrate at 1500 rpm for 30 s. The resultant PbI_2 layer was annealed at 70 °C for 1 min. To form the perovskite, a solution of FAI (90 mg) and MACl (10 mg) in 1 mL IPA was spin-coated onto PbI_2 at 1800 rpm for 30 s. The resultant film was annealed at 150 °C for 15 min.

To deposit $Cs_{0.05}FA_{0.85}MA_{0.1}PbI_3$ (CsFAMAPbI), the precursor solution (1.5 M) was prepared by mixing CsI (19.5 mg), FAI (219.3 mg), MAI (23.8 mg) and PbI_2 (760.7 mg) in 1 mL mixed solvents of DMF:DMSO (4:1 v/v). 12.5 mol% of MACl and 0.08 mol% of ODADI was also added into the precursor. The precursor was spin-coated on the substrate at 1000 rpm for 10 s, and subsequently accelerated to 5000 rpm for 40 s. 5 s before the end of spin-coating, 200 μL ethyl acetate was dripped onto the film as the anti-solvent. The substrates were then annealed at 100 °C for 30 min.

To make the complete device, the fabricated samples were stacked together with their perovskite layers in contact, and were put on the hotplate of a hot-press (N4053-00, NPa SYSTEM Co., Ltd.). The hot-press has two hotplates at the top and bottom which press onto the sample. As shown in Figure S1a, to avoid adhesion of the sample onto the hotplates, polytetrafluoroethylene (PTFE) sheets (TOMBO No. 9001, NICHIAS Corporation) were placed at the top and bottom of the sample. Additionally, a piece of silicone rubber (5 mm

thickness, AS ONE Corporation) was placed between the top hot-plate and the top PTFE sheet to ensure a uniform pressure on the sample. Both the top and bottom hotplates were set at 150 °C, and the pressure was gradually increased to 1.2×10^7 Pa. The maximum pressure was kept for 20 min, after which the pressure was released gradually and the sample was taken off from the hotplate. (Lower temperature and pressure do not consistently result in successful lamination, likely because the perovskite species require a threshold activation energy to become sufficiently mobile for fusion. Additionally, adequate pressure is needed to bring the two films into intimate contact without trapping air bubbles between them.) The same hot-press process conditions were used for fabricating $MAPbI_3$, $FAPbI_3$, and CsFAMAPbI devices. (We would like to note that the process conditions were not fine-tuned in this study, and further optimization could likely lead to improved film quality and, consequently, higher device efficiency.) The two ITO substrates used for the anode and cathode have different patterns so that when stacked, the device area (0.09 cm^2) is defined by the overlapping ITO (Figure S1b). Device characterizations were carried out after the sample has cooled down to room temperature.

During the process above, solution preparation and thin film deposition were carried out in a dry room (at about 24 °C and a dewpoint of −20 °C). Hot-press of the perovskites was carried out in the ambient air without humidity control (about 25 °C and 60 % RH).

Characterizations

J-*V* characteristics were measured in the ambient air using a source meter (Keithley 2420) and a solar simulator (PEC-L01, Peccell Technologies Inc., Xenon white light source) under 1-Sun illumination condition (AM1.5G, 100 mW/cm^2) calibrated by a reference silicon cell (BS-520, Bunkoukeiki Co., Ltd.). The active area is determined by the patterned ITO (Figure S1b) and a photomask (0.09 cm^2). All the *J*-*V* curves were measured with a scan rate of 0.10 V/s without preconditioning. The external quantum efficiency spectrum was acquired in the ambient air by PEC-S20 spectrometer (Peccell Technologies Inc.) which was calibrated by a reference silicon cell (S1337-1010BQ, Bunkoukeiki Co., Ltd.). MPPT measurement was conducted in the ambient air (25 ± 5 °C and 60 ± 20 % relative humidity) using a solar simulator (PEC-L11, Peccell Technologies Inc.) and a source meter (B2901A, Keysight Technologies) operated by a home-made program. No specific measures were taken to control the device temperature.

Electroluminescence was recorded in a dark room using a near-infrared-sensitive camera (CS-W50HD, PLANEX COMMUNICATIONS Inc.). A heat-absorbing filter (S76-HA50, SURUGA SEIKI Co., Ltd.) was placed over the device to block infrared radiation caused by device heating, ensuring that only electroluminescence was detected.

For all the characterizations above, no particular encapsulation was applied to the devices.

XRD was measured by Rigaku MiniFlex 600 with Cu Kα radiation (40 kV, 15 mA). SEM imaging was conducted on JSM-IT710HR (JEOL Ltd.). The Ultraviolet Photoelectron Spectroscopy (UPS) spectra were recorded with Kratos Nova photoelectron spectrometer (Shimadzu Co.) equipped with a He-I source (21.22 eV). The binding energy values were calibrated by measuring a clean Au surface. The sample work function is determined by subtracting the binding energy of the secondary electron cutoff from the photon energy of the UV source (21.22 eV). VBM is obtained by extrapolating the valence band onset at low binding energy. Kelvin-probe force microscopy images were obtained by Shimadzu SPM-9700HT operated in the ambient air. The UV-vis absorption spectra were acquired using Shimadzu UV-1800 Spectrophotometer. The photoluminescence spectra were obtained using JASCO FP-8600 Spectrometer.

Grazing-incidence wide-angle X-ray scattering (GIWAXS) measurements in reciprocal space were performed at the BL19B2 beamline of SPring-8. The perovskite films were irradiated with synchrotron X-rays at a photon energy of 12.39 keV using a Huber

diffractometer, with fixed incident angles of 2.0°. The 2D-GIWAXS patterns were recorded using a two-dimensional area detector (PILATUS 300K). For the analysis of individual diffraction peaks, the integration interval from q_1 to q_2 was appropriately selected. The two-dimensional scattering patterns I_{2D} (q, χ) were further azimuthally integrated to obtain one-dimensional (1D) intensity profiles I_{1D} (q) as follows:

$$I_{1D}(q) = \frac{1}{2\pi}\int_0^{2\pi} I_{2D}(q,\chi)\, d\chi$$

where q is the magnitude of the scattering vector, and χ is the azimuthal angle.

Cost analysis

The precise cost of manufacturing depends on various factors such as specific materials, materials grade, material supplier, purchase quantity, deposition process, device yield, etc. For the purpose of a relative comparison, we simplify the analysis by focusing exclusively on the interfacial materials as the other layers (i.e., the perovskite layer, electrodes and encapsulation materials) can vary across device structures. The cost estimation is based on the amount of materials required to fabricate laboratory-scale devices and the current market price for small quantity purchases as listed on the suppliers' websites, as summarized in Table S2. Although costs at large-scale manufacturing would be significantly lower, the costs associated with fabricating laboratory-scale devices provide a useful rule of thumb.

We compared the cost of the interfacial layers used in the following four types of devices:

- Structure 1: cathode / compact TiO_2 / mesoporous TiO_2 / perovskite / Spiro-MeOTAD / anode
- Structure 2: anode / NiO_x (nanoparticle) / perovskite / Bathocuproine (BCP) / C_{60} / cathode
- Structure 3: anode / PTAA / perovskite / BCP / C_{60} / cathode
- Structure 4 (used in our study): anode / MeO-2PACz / perovskite / C_{60}-SAM / cathode

The calculation assumes that BCP and C_{60} are deposited by thermal evaporation (20 mg per five devices), and all other materials are deposited by spin-coating their solutions (30 uL per device). The total costs of the interfacial materials per device (of about 1 cm^2) are summarized in Table S3. The result shows that the all-SAM structure (Structure 4) used in this study has a lower materials cost, which is about 3% to 10% of that needed for Structure 1-3.

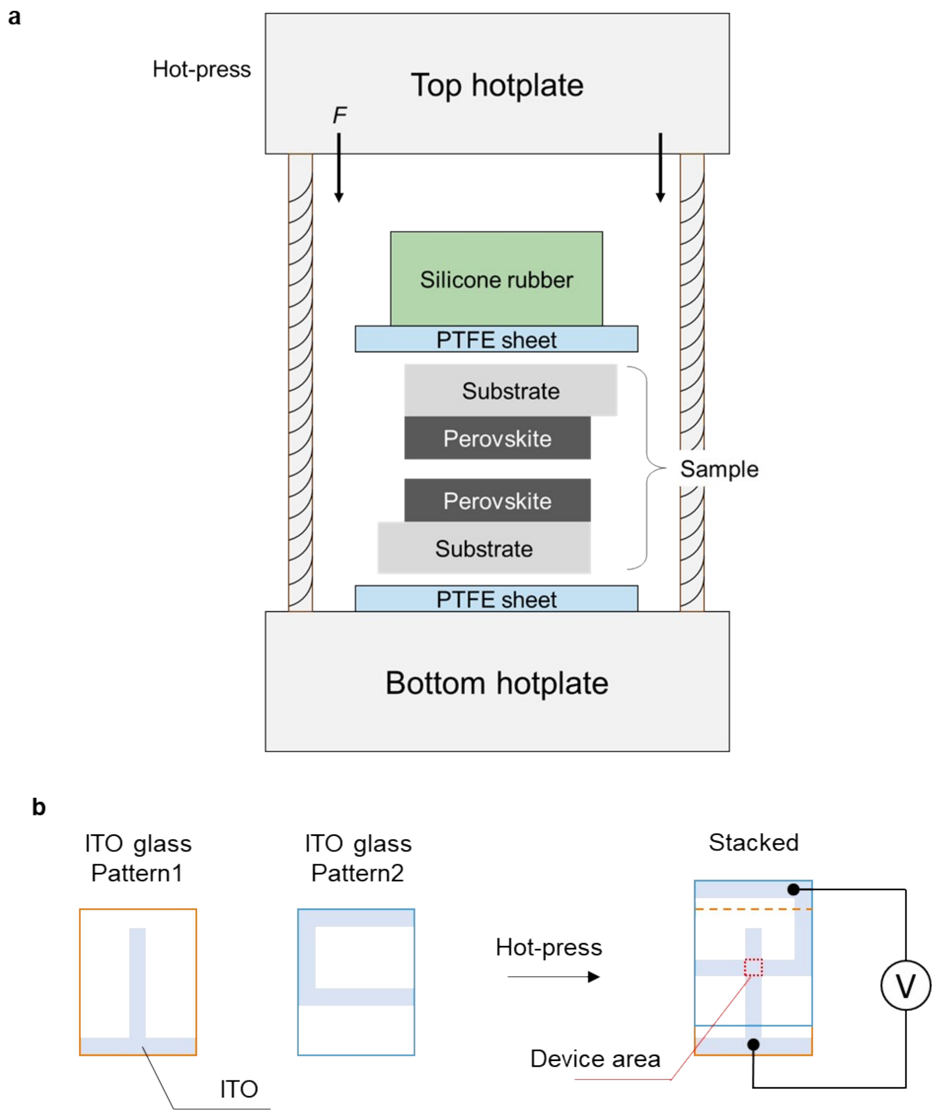

Figure S1 Device fabrication. (a) To fuse two perovskite layers, two perovskite samples were stacked together and placed between the two hotplates in a hot-press. PTFE sheets were placed beneath and on top of the samples, and a piece of silicone rubber was put beneath the top hotplate. **(b)** ITO glass substrates with two types of ITO patterns were used. The stacked samples give a device area of 0.09 cm^2 defined by the overlapping ITO.

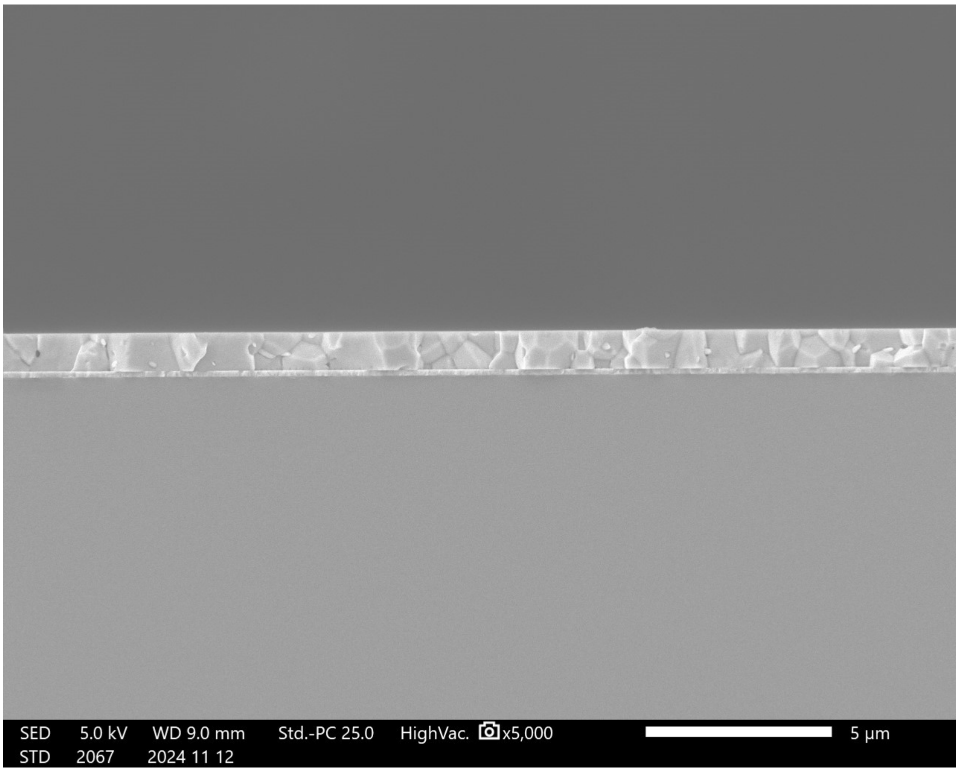

Figure S2 | SEM image. Cross-sectional SEM image of the fused $MAPbI_3$ layer on ITO after hot-pressing, with the upper ITO substrate removed.

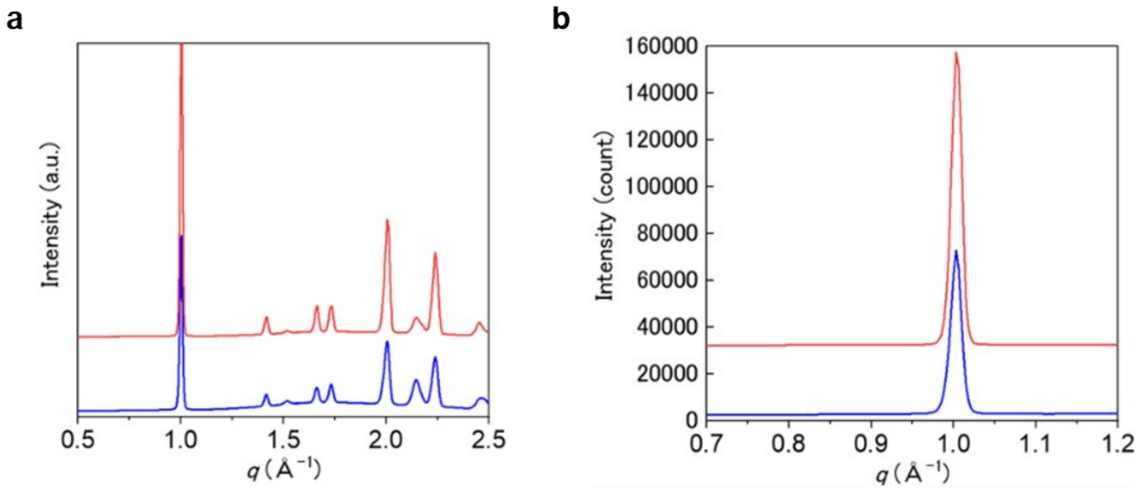

Figure S3 | (a) 1D integrated GIWAXS profiles of $MAPbI_3$, before (blue) and after (red) hot-press. (b) shows a magnified view of the corresponding profiles in the *q* range of 0.7–1.2 $Å^{-1}$.

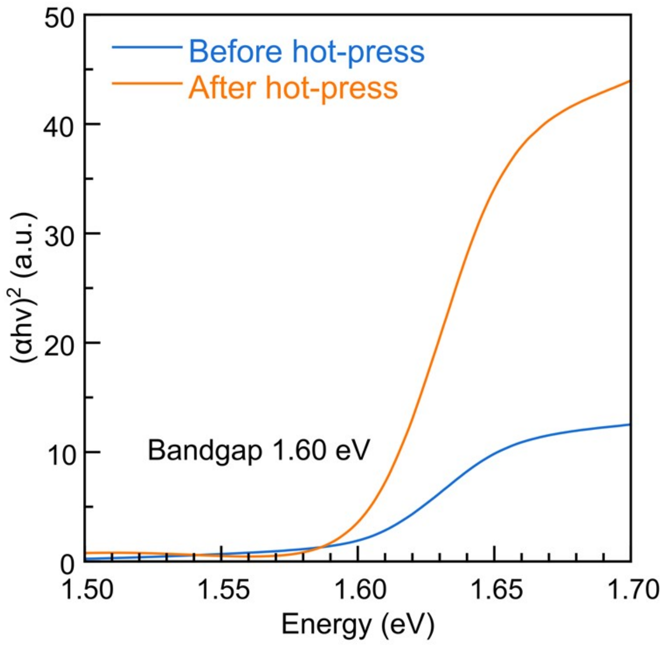

Figure S4 | Tauc plot of the UV-vis absorption spectra of $MAPbI_3$ before and after hot-press.

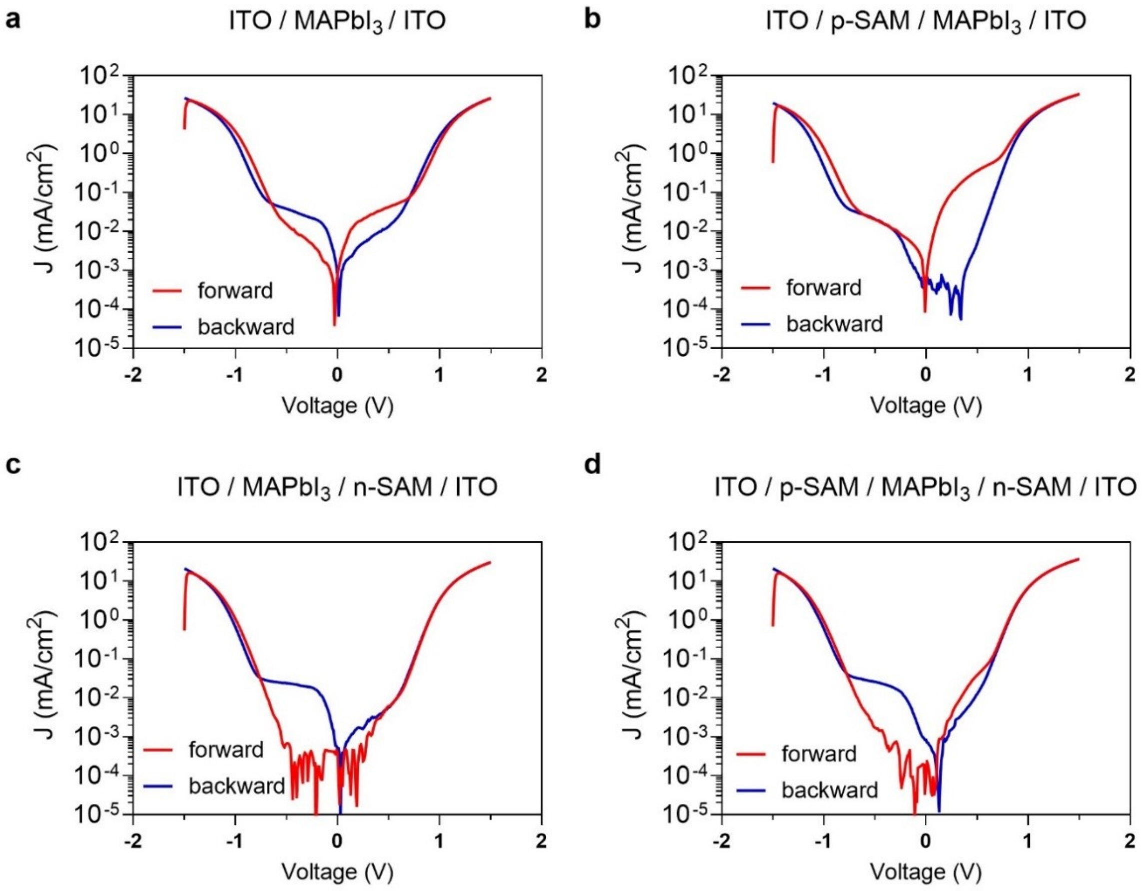

Figure S5 | Dark *J-V* characteristics of the four types of devices under a voltage scan from −1.5 V to 1.5 V and subsequently from 1.5 V to −1.5 V. (a) The device with no SAMs. **(b)** The device incorporating only the p-SAM. **(c)** The device incorporating only the n-SAM. **(d)** The device incorporating both the p-SAM and n-SAM.

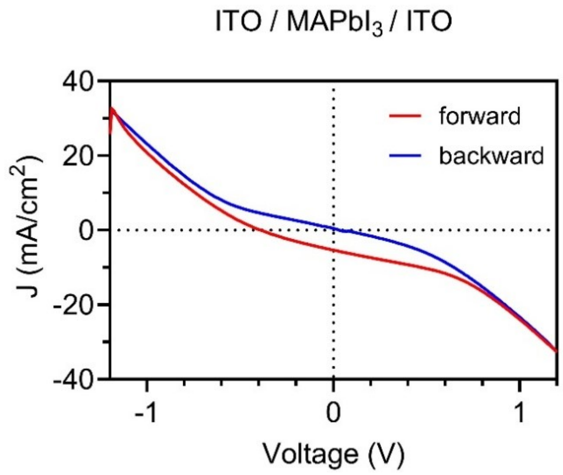

Figure S6 | Photovoltaic *J-V* curves of the device ITO/$MAPbI_3$/ITO under 1-Sun illumination.

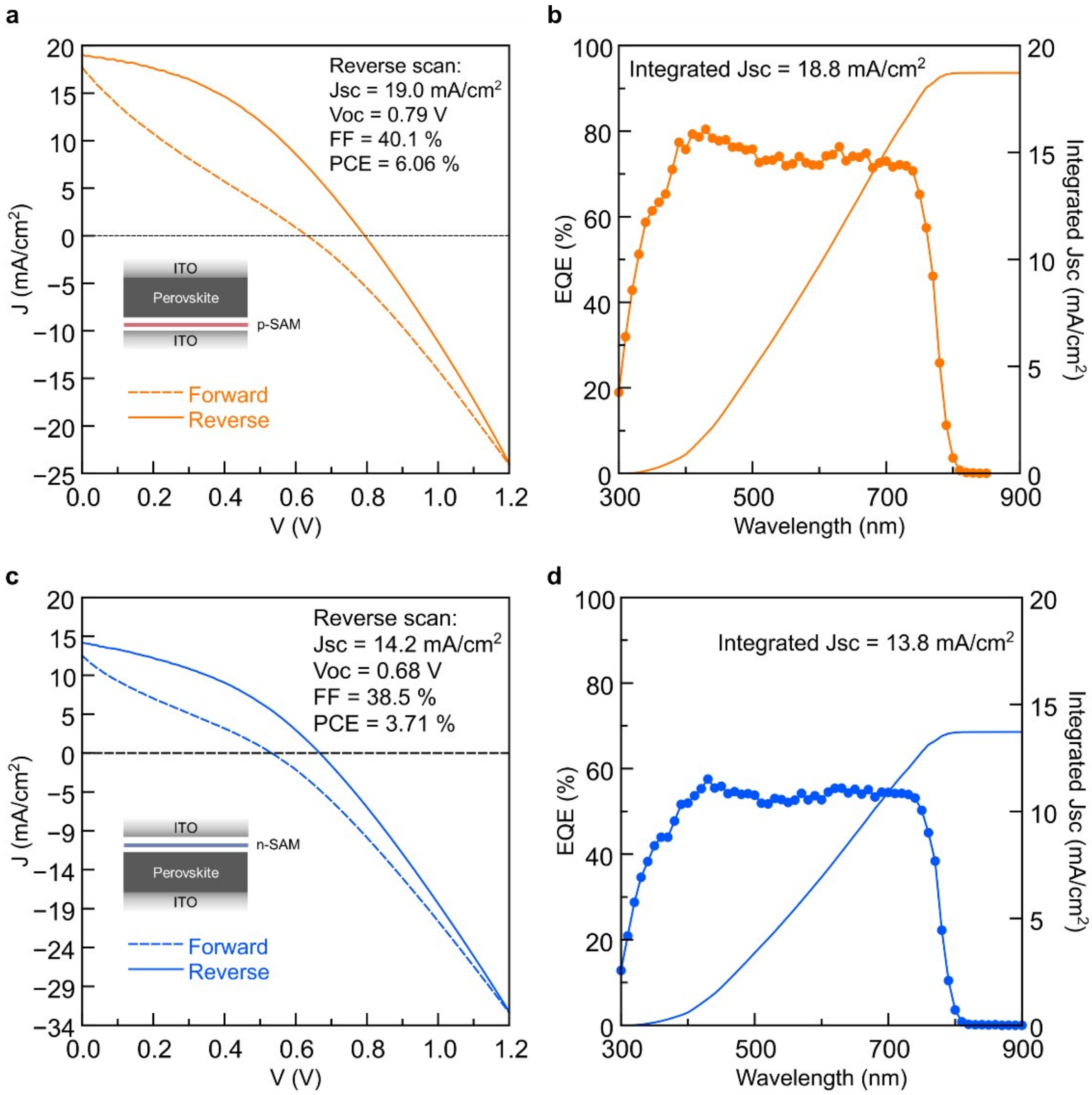

Figure S7 | Photovoltaic performance. (a) *J-V* curve of the p-SAM-only device, **(b)** EQE spectrum and the integrated J_{SC} of the p-SAM-only device, **(c)** *J-V* curve of the n-SAM-only device and **(d)** EQE spectrum and the integrated J_{SC} of the n-SAM-only device.

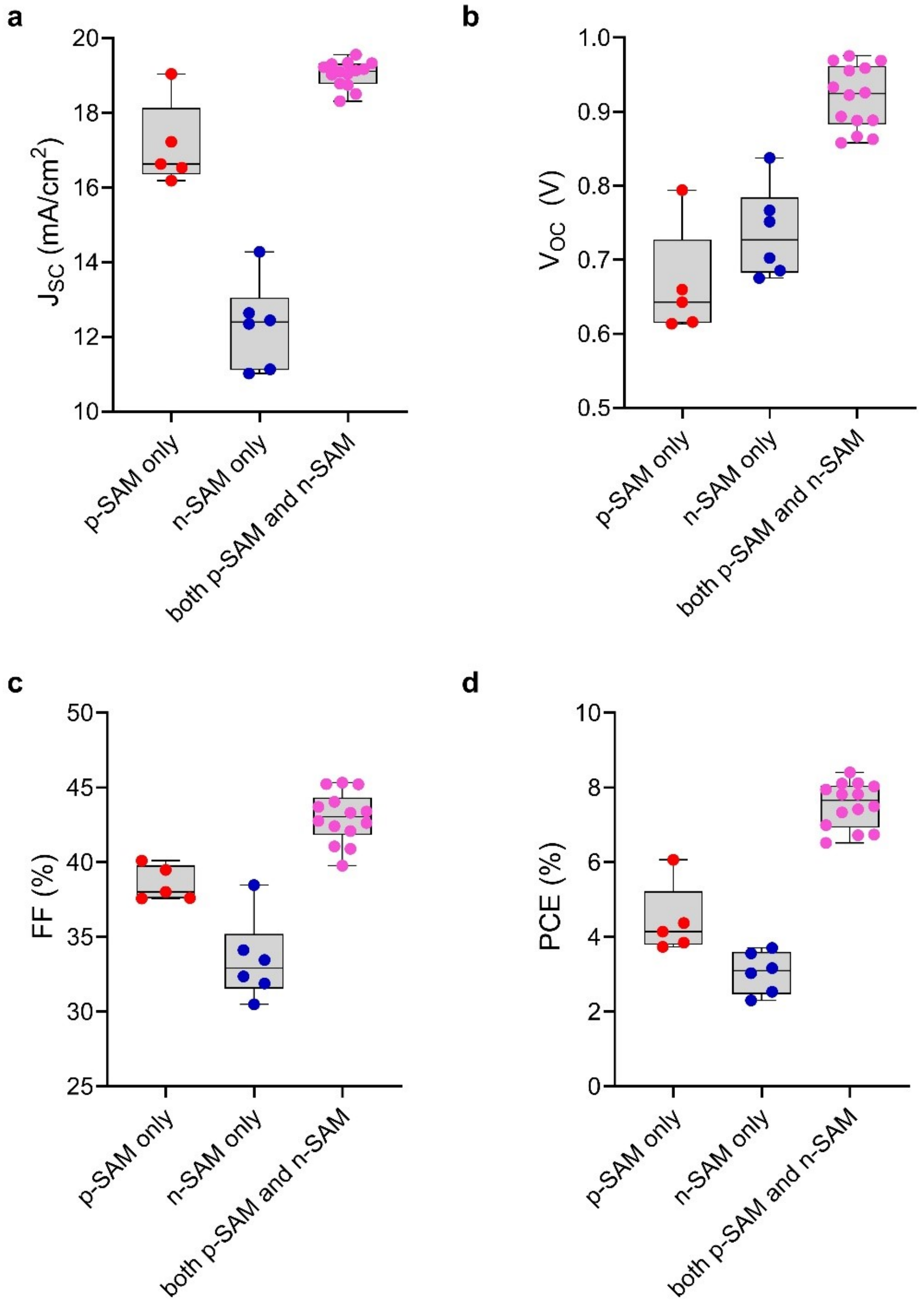

Figure S8 | Statistical distribution of the device performance. (a) J_{SC}, **(b)** V_{OC}, **(c)** FF and **(d)** PCE of the devices incorporating only the p-SAM (ITO/p-SAM/$MAPbI_3$/ITO), incorporating only the n-SAM (ITO/$MAPbI_3$/n-SAM/ITO), and incorporating both the p-SAM and n-SAM (ITO/p-SAM/$MAPbI_3$/n-SAM/ITO). The boxplot shows the minimum, the maximum, the median and the first and third quartiles.

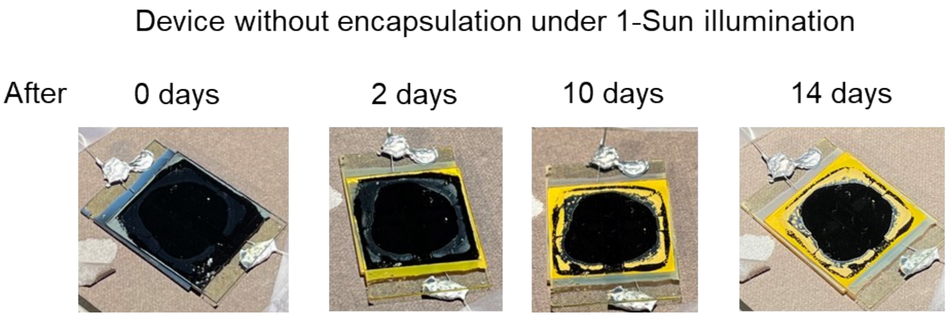

Figure S9 | Degradation of the device (ITO/p-SAM/$MAPbI_3$/n-SAM/ITO) under 1-Sun illumination in a duration of 14 days. No particular encapsulation is added.

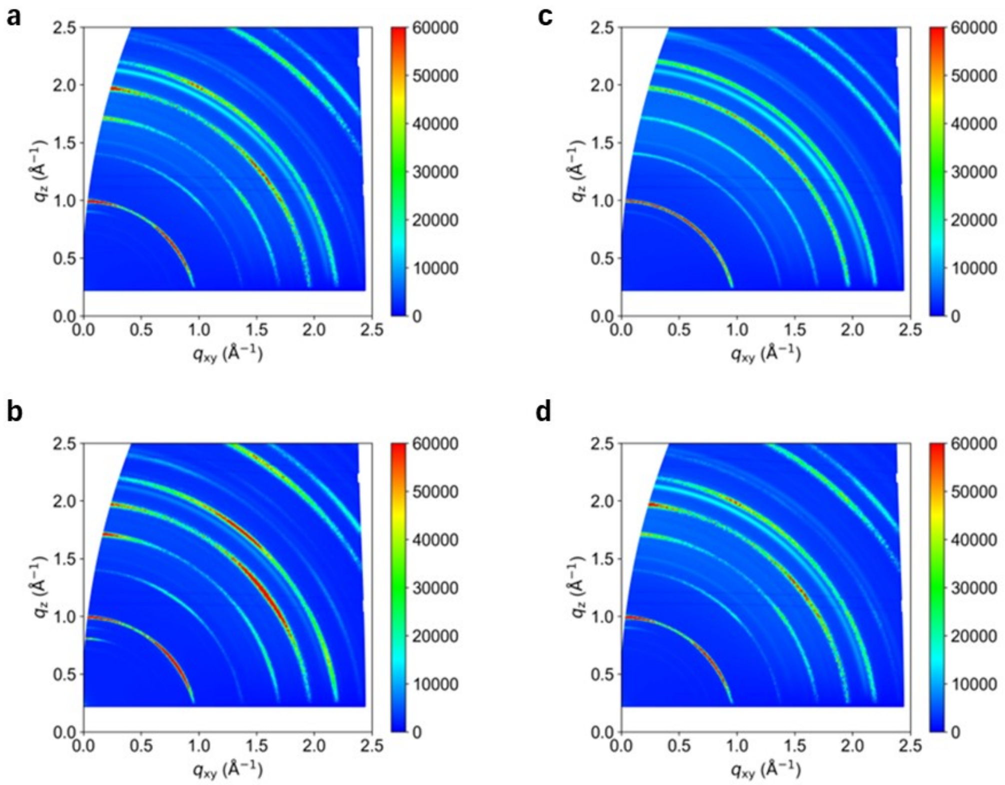

Figure S10 | 2D GIWAXS patterns of $FAPbI_3$ and CsFAMAPbI, measured for each composition before (a,c) and after (b,d) hot-press.

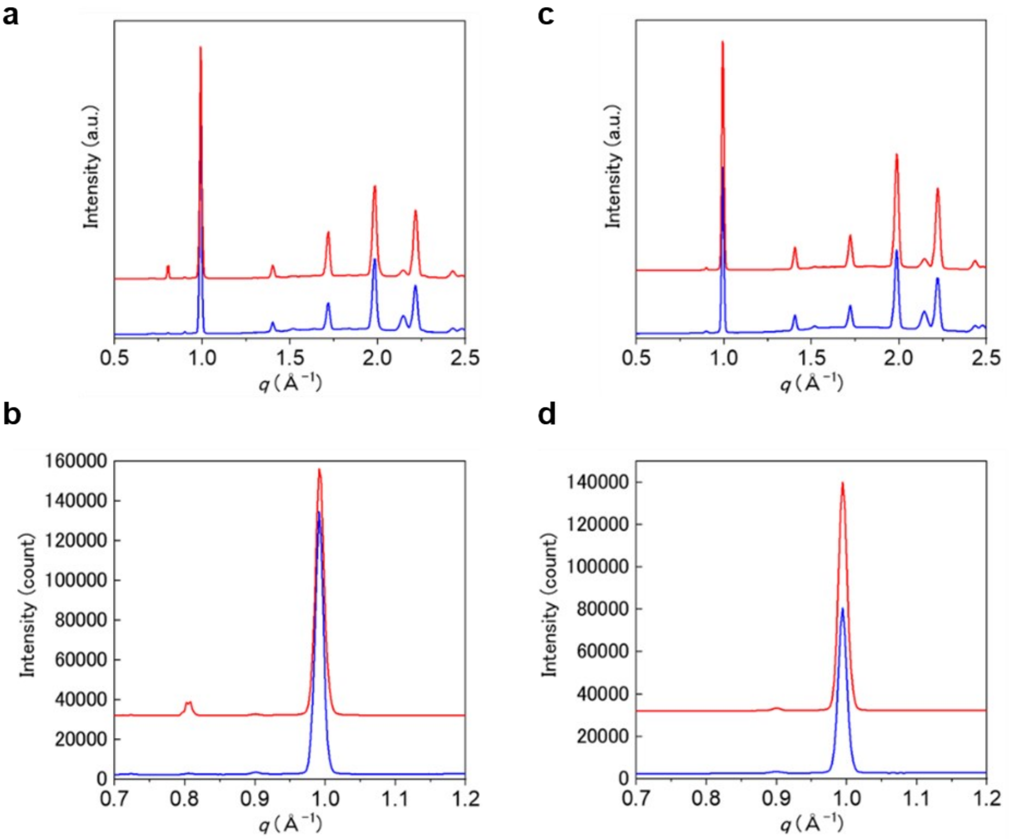

Figure S11 | 1D integrated GIWAXS profiles of (a, b) $FAPbI_3$, (c, d) CsFAMAPbI before (blue) and after (red) hot-press. b and d show magnified views of the corresponding profiles in the q range of 0.7–1.2 $Å^{-1}$.

Table S1 | Full-width at half maximum (FWHM) and crystallite size of $MAPbI_3$, $FAPbI_3$ and CsFAMAPbI determined from GIWAXS analysis of the (002) diffraction peak for $MAPbI_3$ and the (001) diffraction peak for $FAPbI_3$ and CsFAMAPbI.

	Hot-press	FWHM	Crystallite size (nm)
$MAPbI_3$	Before	1.0035	42
	After	1.0035	42
$FAPbI_3$	Before	0.9915	47
	After	0.9915	46
CsFAMAPbI	Before	0.9945	45
	After	0.9945	45

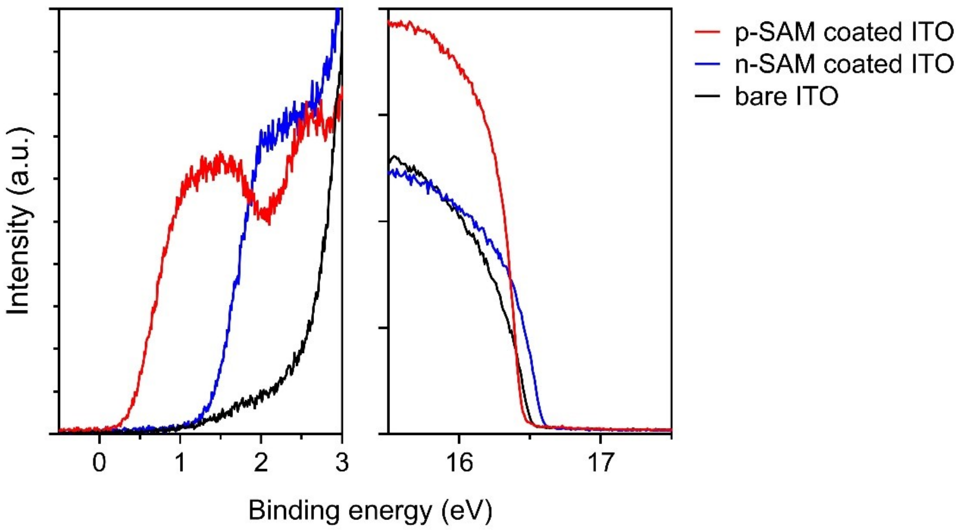

Figure S12 | UPS spectra showing the Fermi edge and secondary electron cut-off. The binding energy (x-axis) and intensity (y-axis) are in the linear scale.

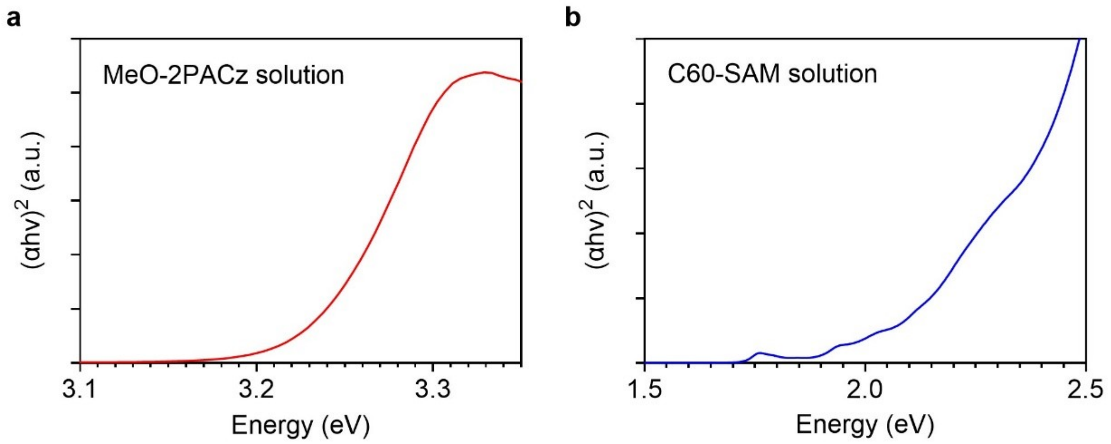

Figure S13 | Tauc plot of the UV-vis absorption spectra. (a) The absorption spectrum of the MeO-2PACz solution. **(b)** The absorption spectrum of the C60-SAM solution.

Table S2 | Current market price (as of January 4th, 2026) of the common interfacial materials.

Chemicals	Supplier	Amount	Price (USD)
Spiro-MeOTAD	Sigma Aldrich	1 g	482
Lithium bis(trifluoromethanesulfonyl)imide (Li-TFSI)	Sigma Aldrich	10 g	78.6
4-tert-Butylpyridine	Sigma Aldrich	25 g	200
Titanium diisopropoxide bis(acetylacetonate) (75 wt. % in isopropanol)	Sigma Aldrich	100 mL	50.2
TiO_2 paste	Greatcell Solar Materials	10 g	57.5
NiO_x nanoparticle ink	Sigma Aldrich	10 mL	131
PTAA	Sigma Aldrich	250 mg	263
C60	Sigma Aldrich	250 mg	183
BCP	Sigma Aldrich	1 g	224
MeO-2PACz	TCI	500 mg	175.39
C60-SAM	Sigma Aldrich	100 mg	396
Acetonitrile (anhydrous)	Sigma Aldrich	100 mL	91.2
CB (anhydrous)	Sigma Aldrich	100 mL	96.2
1-Butanol (anhydrous)	Sigma Aldrich	100 mL	66.8
Toluene	Wako	100 mL	17.54
Ethanol (Super Dehydrated)	Wako	100 mL	17.22
THF (Super Dehydrated)	Wako	100 mL	15.63

Table S3 | Cost of the interfacial materials per device (of about 1 cm^2) with four types of structures.

	Structure 1	Structure 2	Structure 3	Structure 4
Interface 1	compact TiO_2	BCP	BCP	C60-SAM
Interface 2	mesoporous TiO_2	C_{60}	C_{60}	none
Interface 3	Spiro-MeOTAD	NiO_x	PTAA	MeO-2PACz
Cost per device	1.14 USD	4.22 USD	3.89 USD	0.13 USD

Table S4 | Photovoltaic performances of the devices (ITO/p-SAM/perovskite/n-SAM/ITO) incorporating $MAPBI_3$, $FAPbI_3$ and CsFAMAPbI as the perovskite layer.

Perovskite	J_{SC} (mA/cm^2)	V_{OC} (V)	FF (%)	PCE (%)
	Forward scan / Reverse scan			
$MAPbI_3$	18.9 / 19.1	0.96 / 0.98	39.0 / 45.2	7.06 / 8.40
$FAPbI_3$	16.8 / 16.6	1.01 / 1.00	43.3 /39.6	7.34 / 6.53
CsFAMAPbI	18.8 / 18.8	0.93 / 0.92	47.0 / 48.0	8.22 / 8.32
